# Expression of Taste Receptor 2 Subtypes in Human Testis and Sperm

**DOI:** 10.3390/jcm9010264

**Published:** 2020-01-18

**Authors:** Laura Governini, Bianca Semplici, Valentina Pavone, Laura Crifasi, Camilla Marrocco, Vincenzo De Leo, Elisabeth Arlt, Thomas Gudermann, Ingrid Boekhoff, Alice Luddi, Paola Piomboni

**Affiliations:** 1 Department of Molecular and Developmental Medicine, Siena University, 53100 Siena, Italy; laura.governini@unisi.it (L.G.); semplici4@student.unisi.it (B.S.); pavalentina13@gmail.com (V.P.); laura.crifasi@libero.it (L.C.); marrocco@student.unisi.it (C.M.); vincenzo.deleo@unisi.it (V.D.L.); paola.piomboni@unisi.it (P.P.); 2 Walther Straub Institute of Pharmacology and Toxicology, LMU Munich, 80336 Muenchen, Germany; elisabeth.arlt@lrz.uni-muenchen.de (E.A.); thomas.gudermann@lrz.uni-muenchen.de (T.G.); ingrid.boekhoff@lrz.uni-muenchen.de (I.B.)

**Keywords:** human male fertility, bitter taste receptors, human sperm, human testis, GPCRs, α-gustducin, α-transducin, TRPM5

## Abstract

Taste receptors (TASRs) are expressed not only in the oral cavity but also throughout the body, thus suggesting that they may play different roles in organ systems beyond the tongue. Recent studies showed the expression of several TASRs in mammalian testis and sperm, indicating an involvement of these receptors in male gametogenesis and fertility. This notion is supported by an impaired reproductive phenotype of mouse carrying targeted deletion of taste receptor genes, as well as by a significant correlation between human semen parameters and specific polymorphisms of taste receptor genes. To better understand the biological and thus clinical significance of these receptors for human reproduction, we analyzed the expression of several members of the TAS2Rs family of bitter receptors in human testis and in ejaculated sperm before and after in vitro selection and capacitation. Our results provide evidence for the expression of *TAS2R* genes, with TAS2R14 being the most expressed bitter receptor subtype in both testis tissue and sperm cells, respectively. In addition, it was observed that in vitro capacitation significantly affects both the expression and the subcellular localization of these receptors in isolated spermatozoa. Interestingly, α-gustducin and α-transducin, two Gα subunits expressed in taste buds on the tongue, are also expressed in human spermatozoa; moreover, a subcellular redistribution of both G protein α-subunits to different sub-compartments of sperm was registered upon in vitro capacitation. Finally, we shed light on the possible downstream transduction pathway initiated upon taste receptor activation in the male reproductive system. Performing ultrasensitive droplets digital PCR assays to quantify RNA copy numbers of a distinct gene, we found a significant correlation between the expression of TAS2Rs and TRPM5 (*r* = 0.87), the cation channel involved in bitter but also sweet and umami taste transduction in taste buds on the tongue. Even if further studies are needed to clarify the precise functional role of taste receptors for successful reproduction, the presented findings significantly extend our knowledge of the biological role of TAS2Rs for human male fertility.

## 1. Introduction

The designation “taste receptors” (TASRs) initially derives from the first identification of TASRs in small clusters of specialized epithelial cells on the tongue, called taste buds [1]. The term “taste” is usually used to describe sensations arising from the oral cavity. However, numerous studies published in recent years point the attention on the expression of TASRs throughout the body, both in humans and in animals [2]. Their expression has been reported, among others, in the respiratory [3] and digestive [4] systems, in brain [5], as well as in testis [6,7,8,9]. Therefore, a physiological role of taste receptors beyond the tongue has been hypothesized, even though the functional role of this extraoral expression has not yet been definitely clarified.

The gustatory system can discriminate five distinct taste modalities: sweet, bitter, salty, sour, and umami. Salty and sour tastes are both directly sensed by ion channels [10,11], whereas umami, sweet, and bitter taste stimuli are recognized by G protein-coupled receptors (GPCRs) [1,12,13]. Two different families of taste GPCRs have been identified: Type 1 Taste Receptors (TAS1Rs) and Type 2 Taste Receptors (TAS2Rs) [14,15]. Importantly, TAS1Rs are responsible for perceiving sweet compounds and umami, defined as the taste of monosodium glutamate [16,17], whereas TAS2Rs are operative in detecting the vast variety of bitter molecules, both natural and synthetic [15,18,19,20].

TAS1Rs and TAS2Rs generally activate the same signal transduction pathway: when tastants bind to its specific receptor, an associated heterotrimeric G protein, composed by an α and βγ subunit, is activated. The primarily taste-specific G protein α-subunit gustducin was first identified in 1992 in taste cells of the tongue [21], but Fehr and coworkers demonstrated also an expression of this signaling protein in testis tissue and mature spermatozoa of different mammalian species [22]. Li and Zhou [7] confirmed the presence of α-gustducin in the mouse testis, with a wide distribution from the basal to the luminal compartment of the seminiferous tubules within the testis.

Beside α-gustducin, α-transducin, a G protein α-subunit with a high degree of sequence homology to gustducin naturally expressed in vertebrate retina rods and cones, was found to be also expressed in taste buds [23,24]. Importantly, due to the high sequence homology between both α-subtypes, it has been suggested that both G proteins show a similar or even equivalent mode of action.

The presence of taste receptors but also proteins, specifically involved in TASR signal transduction, paved the way for subsequent physiologically based studies in the male reproductive system, in order to uncover the concrete expression profile and furthermore, the possible involvement of taste receptors in male fertility. Indeed, it was found that the two subunits of the umami taste receptor dimer (TAS1R2/TAS1R3) are expressed in mouse and human spermatozoa [8]. Moreover, it was demonstrated that silencing or reduced expression of TAS1Rs significantly affects sperm production and thus fertility [7,9,22,25]. For bitter taste receptors, Xu et al. showed that a high percentage of the 35 identified mouse *TAS2R* genes are highly expressed in the testis, particularly in postmeiotic germ cells [26], thus suggesting an involvement of Tas2Rs in regulating the communication between germ cells and the testicular microenvironment.

TAS2R’s activation instantly actuates an innate aversion response, which is important because many bitter tastants are potentially toxic. Since TAS2R receptors are thus able to provide cautioning signals, the ingestion of noxious compounds in spoiled food can be prevented [27]. However, some bitter substances could have healthy benefits and stimulate a sense of pleasure or support guiding food choice [28,29]. In this context, it is important to mention that bitter taste cells in the mouth do not uniformly express all 25 human bitter taste receptor genes, but in contrast represent a heterogeneous cell population characterized by a distinct expression profile of individual TAS2R subtype [30]. The thus derived ability of individual taste cells to discriminate between different bitter stimuli along with the reported expression of taste receptors on motile cilia [3] let us hypothesize that these receptors may present specialized molecular sensors fundamental in sperm chemosensation and/or guidance.

To the best of our knowledge, no studies have addressed the expression of TAS2Rs in the human male reproductive system. Here, we have investigated the expression of selected individual receptor subtypes belonging to the TAS2R family, namely TAS2R3, TAS2R4, TAS2R14, TAS2R19, and TAS2R43, in human testis and ejaculated spermatozoa. We have also considered the expression of molecules involved in signal transduction processes elicited upon activation of this class of receptors.

## 2. Materials and Method

### 2.1. Patients

This study was conducted on a total of 15 Caucasian males undergoing semen evaluation at the Unit of Medically Assisted Reproduction, at Siena University Hospital. A comprehensive clinical history of patients was obtained, and possible causes of male infertility such as varicocele, cryptorchidism, or endocrine disorders were excluded. The median age of the patients was 33 years (22–42).

Testis samples were obtained from six patients undergoing diagnostic testicular biopsy for obstructive azoospermia. None of these patients received chemotherapy, radiotherapy, or hormonal treatment. Upon testicular biopsies, around 5 mm^3^ of tissue was cryopreserved in RNAlater (Qiagen, Hilden, Germany) and stored at −80 °C. All participants have signed a written informed consent, and the study protocol was approved by the Ethic Committee of the Siena University Hospital (approval ID: CEASVE 191113).

### 2.2. Sample Collection and Analysis

The semen samples were obtained by masturbation after 3–5 days of sexual abstinence. Standard semen analysis was performed according to WHO protocol [31], and all subjects were characterized for main sperm parameters, namely concentration, morphology, progressive, and total motility.

The samples were then divided into two aliquots, in order to collect sperm before and after in vitro capacitation. To this end, one aliquot was centrifuged at 400 rcf for 10 min, washed in phosphate-buffered saline (PBS), and then centrifuged at 400 rcf (relative centrifugal force) for 10 min, for three times. The second aliquot of seminal fluid was stratified on a gradient of 80% and 40% Pure Sperm (Nidacon, Mölndal, Sweden) and centrifuged at 400 rcf for 20 min. The pellet was recovered from the bottom of the conical tube and washed twice with Sperm washing medium (FUJIFILM Irvine Scientific, Santa Ana, CA, USA). The sperm were capacitated according to Stendardi et al. [32]. The sperm pellets before and after gradient selection and capacitation were resuspended in Trizol (TRI Reagent^®^ Sigma-Aldrich, Burlington, MA, USA) for gene expression studies, or in RIPA buffer (Sigma-Aldrich, Burlington, MA, USA) for protein analysis and stored at −80°C; for immunofluorescence analysis the samples were washed in PBS and then immediately processed as described below.

### 2.3. RNA Extraction and Droplets Digital PCR Assay

Total RNA was extracted from ejaculated sperm and from tissue samples as follows. Briefly, RNA isolation from sperm resuspended in Trizol was performed using TRI Reagent^®^ Sigma protocol (Sigma, MA, USA), according to the manufacturer’s instruction. RNA extraction from frozen testis biopsies was obtained by disrupting the tissue that was then homogenized and immediately processed for RNA extraction using the RNeasy Protect Mini kit, according to the manufacturer’s instructions (Qiagen, Hilden, Germany).

RNA quantity was assessed using an ND-1000 Nanodrop Spectrometer (Thermo Fisher Scientific, Wilmington, DE, USA). Complementary DNA (cDNA) was generated from 200 ng of each RNA sample using the iScript™ gDNA Clear cDNA Synthesis Kit (Bio-Rad, Hercules, CA, USA). Gene expression was evaluated using specific EvaGreen assays (Table 1), using the Bio-Rad’s QX200 ddPCR System (Bio-Rad, CA, USA).

Droplets digital polymerase chain reaction (ddPCR) was performed in a total volume of 22 μL, containing 11 μL 2X QX200 ddPCR EvaGreen Supermix (Bio-Rad, CA, USA), 1.1 μL PrimePCR EvaGreen Assays, 8 μL RNase-free sterile water, and 2.5 μL diluted 1:10 cDNA. The mixture was added to the DG8 cartridge, followed by the loading of 70 μL of droplet generation oil for EvaGreen (Bio-Rad), using an automated Droplet Generator (Bio-Rad, CA, USA). Then, 40 µL of generated droplets were transferred into a 96-well PCR plate and heat-sealed with pierceable foil by using a PX1 PCR Plate Sealer (Bio-Rad, CA, USA), for 3 s at 175 °C twice before thermal cycling, and then placed in a thermal cycler (T100 Thermal Cycler, Bio-Rad, CA, USA). The cycling conditions were as follows: 5 min at 95 °C, 30 s at 96 °C, 1 min at 58 °C, 5 min at 4 °C, and 5 min at 90 °C. After PCR, the 96-well PCR plate was loaded into the QX200 Droplet Reader (Bio-Rad, CA, USA), to identify the fluorescence intensity of each droplet for EvaGreen fluorophore. Data were analyzed using the QuantaSoftTM Analysis Pro software, version 1.0 (Bio-Rad, CA, USA). A threshold line was employed to discriminate positive and negative droplets. The Poisson statistics were applied to calculate the absolute concentration of each target gene in copies/μL. The reference genes were used to normalize RNA amount, obtaining a final value of relative gene expression, expressed as normalized sample amount (NSA).

### 2.4. Western Blot Analysis

An amount of 50 μg of total proteins from each sample were separated according to their molecular weight on 10% SDS-PAGE gel following the method described by Laemmli [33]. The electrophoretic run has been conducted in a buffer containing Tris 0.6%, SDS 0.1%, glycine 2.88% at 25 mA/gel, using the Cell Mini Protean (BioRad Microsciences). Proteins separated by SDS-PAGE were electroblotted from polyacrylamide gels to nitrocellulose in a mini Trans-Blot apparatus (Bio-Rad, CA, USA). Membranes were subsequently blocked with 5% nonfat dry milk in TBS (Tris-buffered saline, 10 mM Tris-HCl, pH 7.5 and 0.15 M NaCl) for 1 h at room temperature, and then they were probed overnight at 4 °C with primary polyclonal antibody as reported in Supplementary Table S1, diluted in 1% nonfat dry milk/TTBS (TBS containing 0.2% Tween 20). After washing, membranes were incubated for 1 h with the appropriated horseradish peroxidase (HRP)-conjugated secondary antibody. The same nitrocellulose membranes were also incubated with an anti-GAPDH (Glyceraldehyde-3-phosphate dehydrogenase) antibody, followed by the appropriate secondary antibody. The chemiluminescence signals were captured using an XRS instrument ChemiDoc (Bio-Rad Microsciences, Hemel Hempstead, UK). Images were then processed with the use of Quantity One 4.5.7 and PDQuest 7.4.0 software (Bio-Rad Microsciences) for spot identification and quantification as pixel/mm^2^.

### 2.5. Immunofluorescence

Sperm samples before and after in vitro selection and capacitation were washed in PBS and centrifuged at 500 rcf for 10 min. The sperm pellet fractions were resuspended in PBS, smeared on glass slides, and fixed in (with) 4% paraformaldehyde in PBS. Glass slides were immunolabeled using an indirect procedure. All incubations were performed in blocking solution containing 5% goat serum. Specificity of immunostaining was confirmed by both omission of primary antibody and staining of sections with unrelated antibodies. For single staining, the slides were incubated for 1 h at RT with the primary antibody (Supplementary Table S1). Glass slides were then washed in PBS, and the bound antibodies were visualized by incubation with a secondary antibody (Supplementary Table S1). After that, sperm were incubated with 30 µg/mL FITC–PSA (Pisum sativum agglutinin) in PBS for 30 min and washed again with a stream of distilled water for 10 min. Sperm nuclei were finally counterstained with DAPI (6-diamino-2phenylindole) for 30 min at RT. After washing in PBS, the slides were mounted with an antifade solution, in order to avoid fading of fluorescence, and immediately observed with the fluorescence microscope LEICA CTR6500HS.

### 2.6. Statistical Analysis

Statistical analysis was performed using the GraphPad Prism 5.0 (GraphPad Software, San Diego, CA, USA). Statistical significance was evaluated by using nonparametric tests. Differences among groups of data were tested by the Kruskal–Wallis test or the Mann–Whitney test. Statistical significance was set at *p* < 0.05. Correlation was determined by using Spearman’s correlation analysis.

## 3. Results

### 3.1. Taste Receptor Expression in Human Sperm and Testis Tissue

ddPCR was carried out in order to determine the relative expression of selected taste genes belonging to the TAS2R family in human sperm collected from 15 normozoospermic men, whose main sperm parameters resulted within the range of normality, according to WHO criteria [31]: sperm concentration was 32.7 × 10^6^ (range 21 × 10^6^–47 × 10^6^), total sperm motility was 53% (range 36–68%), and typical morphology was 7% (range 4–10%).

After total RNA isolation from ejaculated sperm, the expression of *TAS2R3*, *TAS2R4*, *TAS2R14*, *TAS2R19,* and *TAS2R43* was measured and normalized to four reference genes (*PPIB, GAPDH, ACT-B,* and *B2M)*.

**Figure 1:**
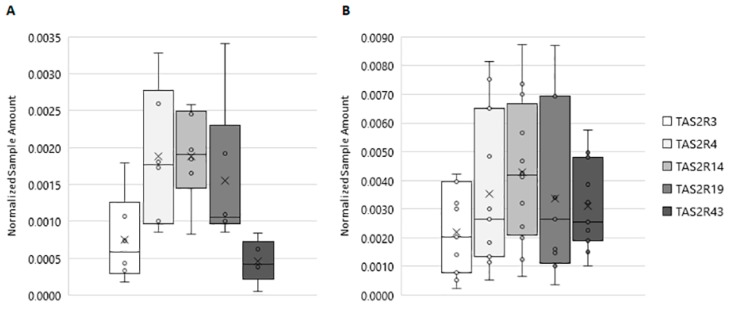
Expression of *TAS2Rs* in human testis and in ejaculated sperm. (**A**) *TAS2Rs* normalized sample amount in human testis. (**B**) *TAS2Rs* normalized sample amount in human sperm. Graphical diagrams are plotted as box–whisker plots, where boxes show the interquartile range with median and mean values, and whiskers represent min and max confidence intervals. Number of analyzed samples: testis: 6, sperm: 15.

As showed in Figure 1A, all investigated *TAS2R* genes are expressed in human testis, although different expression levels were detectable. In ejaculated sperm samples, *TAS2Rs* showed the same expression profile as detected for testis tissue, although the Normalized Sample Amount (NSA) was found to be significantly higher (Figure 1B). Moreover, an inter-individual variation could be seen for single TAS2R subtypes; in fact, we detected the expression of *TAS2R3*, *TAS2R4*, *TAS2R19,* and *TAS2R43* in about 73% of semen samples (11 samples out of 15), whereas *TAS2R14* was detectable in 80% of the probes (12 samples out of 15). This latter gene was found to be expressed in ejaculated sperm at the highest level, with a NSA of 0.0043.

**Figure 2:**
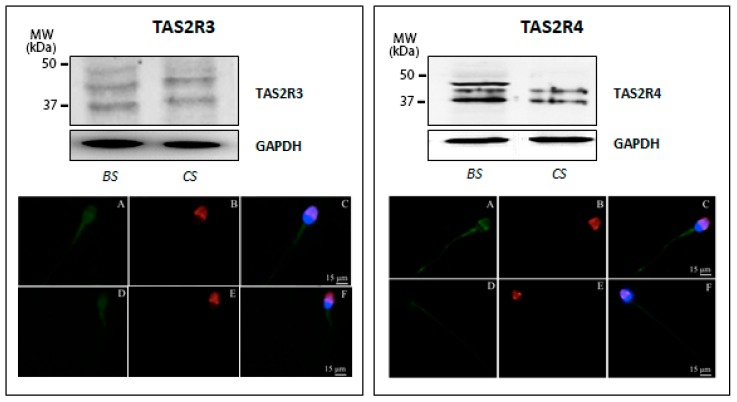
Expression of TAS2R3 (left panel) and TAS2R4 (right panel) in human ejaculated sperm. (**Top**) Western blot of TAS2R3 and TAS2R4 in ejaculated sperm before (BS) and after in vitro capacitation (CS). Equal protein loading of the two sperm preparations was verified using the housekeeping GAPDH. (**Bottom**) Representative micrographs (from three independent experiments) of immunofluorescence staining of TAS2Rs (green), the acrosomal lectin marker PSA (red) and the fluorescent DNA binding dye DAPI (6-diamino-2phenylindole, blue) in ejaculated sperm before (**A**–**C**) and after in vitro capacitation (**D**–**F**). Micrographs in C and F are composed by an overlay of the three fluorescent channels (TAS2Rs green; PSA, red; DAPI, blue); Scale bar = 15 μm.

In order to confirm data from the presented molecular studies, we next analyzed TAS2R expression at the protein level by examining protein extracts prepared from human sperm before and after in vitro capacitation using the Western blot technique. Western blot analysis confirmed the low expression of TAS2R3 already highlighted by ddPCR and revealed the presence of three distinct bands with molecular weights ranging from 35 to 50 kDa (calculated molecular mass of 36 kDa). Although not definitively clarified for sperm cells, visible immunoreactive bands larger than the predicted molecular size of the receptor may account for the previously described glycosylation of TAS2Rs [34]. Interestingly, immunoreactivity visualizing the receptor with the highest molecular weight (ca. 50 kDa) was only detectable in uncapacitated sperm, whereas capacitated sperm only showed the expression of the two smaller proteins (Figure 2, left panel). To evaluate whether the glycosylation status also affects subcellular localization upon capacitation, immunofluorescence analysis of sperm incubated with primary antibodies generated against individual TAS2R subtypes was performed. The results demonstrated in Figure 2 (left panel) illustrate a faint TAS2R3-derived staining at the equatorial segment of the head and the midpiece segment of the tail, in both uncapacitated and capacitated sperm, suggesting that the distribution of this TAS2R subtype did not change upon secondary sperm maturation.

As regard to TAS2R4, an immunoreactive band with the calculated molecular mass of 34 kDa was visible in capacitated sperm; in addition, two additional discrete bands with a higher molecular weight were detectable in the freshly prepared sperm suspension, which possibly also reflect a different degree of receptor glycosylation. Remarkably, in vitro capacitation led to a change in the expression pattern of TAS2R4, accompanied by a complete disappearance of the immunoreactive band with the highest molecular mass (Figure 2, right panel). Indirect immunofluorescence analyses confirmed TAS2R4 expression in human sperm and depict that TAS2R4 was mainly present in the equatorial segment of the head and the midpiece and end piece of the tail (Figure 2, lower micrographs in the right panel).

**Figure 3:**
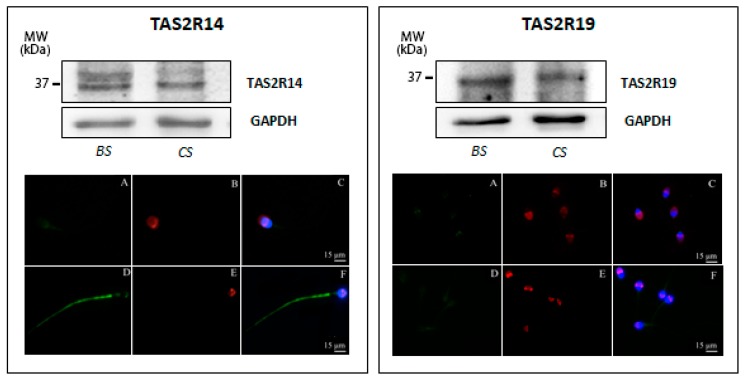
Expression of TAS2R14 (left panel) and TAS2R19 (right panel) in human ejaculated sperm. (**Top**) Western blot of TAS2R14 and TAS2R19 in ejaculated sperm before (BS) and after in vitro capacitation (CS). Equal protein loading of lanes of both sperm preparations was confirmed using an antibody recognizing GAPDH. (**Bottom**) Representative images (from three independent experiments) of immunofluorescence staining of TAS2Rs (green), PSA (red), and DAPI (blue) in ejaculated sperm in basal conditions (**A**–**C**) and after in vitro capacitation (**D**–**F**). Scale bar = 15 μm.

The TAS2R14 protein at the predicted molecular mass of 36 kDa (GenBank accession no. NP_076411, 36029 Dalton) was detected in both basal and capacitated sperm; however, the additional immunopositive band with a higher molecular weight (−45 kDa) was mainly present in uncapacitated sperm, whereas capacitated sperm only show a very faint 45 kDa derived staining (Figure 3, left panel). Subsequent immunofluorescence analysis of TAS2R14 illustrates a faint TAS2R14-derived labeling in the area of the postacrosomal region and a weak but restricted staining of the mitochondria reach midpiece region of the sperm tail of uncapacitated sperm (Figure 3, left panel, A–C); in contrast, in capacitated sperm, a bright staining of the whole tail was evident, as well as a very faint staining in the acrosomal region and the postnuclear region (Figure 3, left panel, D–F). These results uncover that TAS2R14, highly expressed in human spermatozoa, indeed shows a redistribution upon capacitation.

Next, expression of TAS2R19 was examined. Both freshly prepared uncapacitated and capacitated sperm express TAS2R19, as demonstrated by the presence of a 33 kDa immunopositive band (Figure 3, right panel), corresponding to the predicted published molecular size of this receptor (GenBank accession no. NP 795369, 33777 Dalton). This protein is localized to the basal region of the head and within the neck region; capacitation, as well as acrosomal reaction, seems not to affect its subcellular localization (Figure 3, right panel).

**Figure 4:**
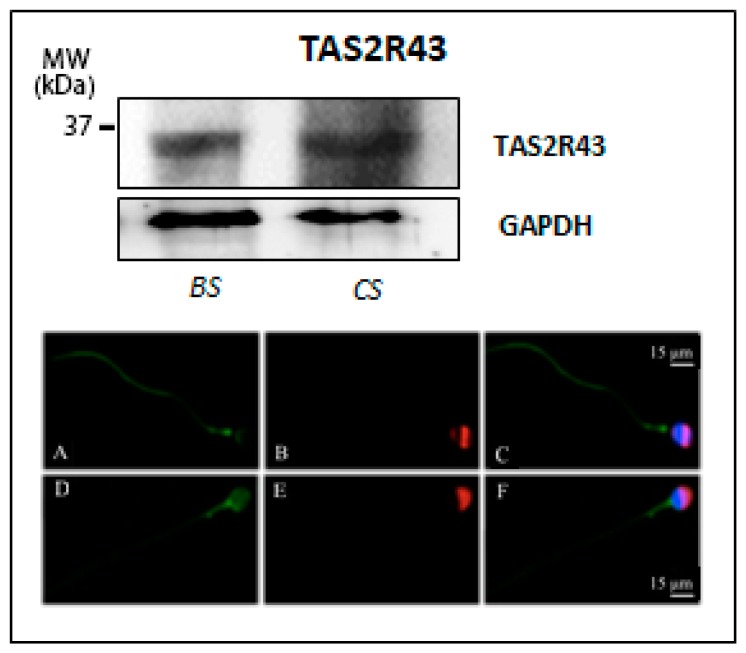
Expression of TAS2R43 in human ejaculated sperm. (**Top**) Western blot of TAS2R43 in ejaculated sperm before (BS) and after in vitro capacitation (CS). GAPDH was immunodetected to control equal loading per lane of each sperm preparation. (**Bottom**) Representative micrographs (from three independent experiments) of immunofluorescence staining of TAS2R43 (green), PSA (red), and DAPI (blue) in ejaculated sperm in uncapacitated sperm (**A**–**C**) and after in vitro capacitation of male gametes (**D**–**F**). Scale bar = 15 μm.

Finally, as shown in Figure 4, an evident band of about 33 kDa, corresponding to the predicted molecular weight of TAS2R43 (GenBank accession no. NP_795365, 35468 Dalton), was visualized, which was present in both uncapacitated and capacitated sperm. TAS2R43 immunolocalization in uncapacitated spermatozoa was characterized by a positive labelling of the pericentriolar region and a staining along the whole sperm tail. After the capacitation process, TAS2R43 staining appears more diffuse in the entire head and in the midpiece region of the flagellum, while it disappears from the principal piece of the tail (Figure 4, micrographs in the bottom), suggesting that capacitation seems also to affect subcellular localization of this bitter taste receptor subtype.

After immunoblotting of TAS2Rs members, a densitometric analysis was carried out, and the obtained data, which are summarized in Supplementary Figure S1, confirm the different expression levels already highlighted by ddPCR.

### 3.2. Mechanisms Underlying Taste Transduction Processes in Human Sperm

**Figure 5:**
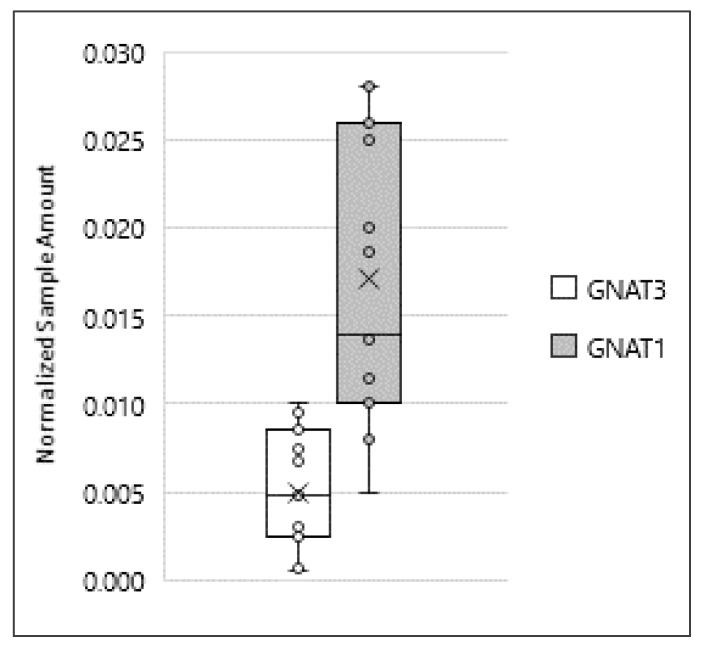
Expression of *GNAT3* and *GNAT1* in ejaculated human sperm. Graphical diagram is plotted as box–whisker plots, where boxes show the interquartile range with median and mean values, and whiskers represent min and max confidence intervals. Number of analyzed samples: testis: 6, sperm: 15.

In taste cells of the tongue, all TAS2Rs receptors generally activate the same downstream signaling effectors, starting predominantly with the activation of the Gα subunit gustducin [35]. Many studies reported gustducin expression in mouse testis and sperm, with only one pointing out its expression in human sperm [19,22,24,36]. α-gustducin is coded by the gene *GNAT3*, which displays considerable sequence homology to *GNAT1*, the gene coding for α-transducin, which is also expressed in taste buds [24,36]. Our data, obtained by ddPCR, demonstrated for the first time that mRNA for these two Gα subunits are detectable in human sperm, with transducin being present at a higher level than gustducin (Figure 5).

**Figure 6:**
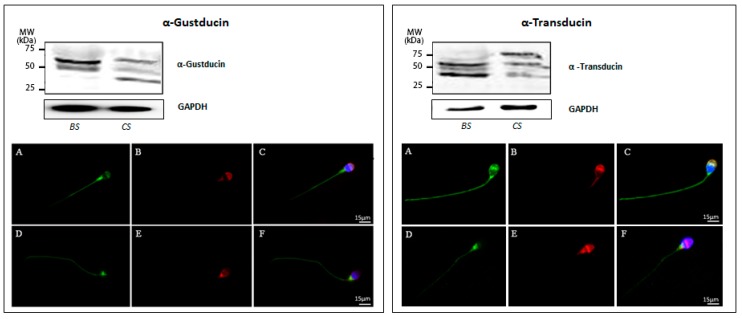
Expression of α-gustducin (left panel) and α-transducin (right panel) in human ejaculated sperm. (**Top**) Western blot of α-gustducin and α-transducin in ejaculated sperm before (BS) and after in vitro capacitation (CS). GAPDH was immunodetected to control equal loading per lane for the two examined sperm suspensions. (**Bottom**) Representative images of immunofluorescence staining (from three independent experiments) of α-gustducin and α-transducin (green), PSA (red), and DAPI (blue) in ejaculated sperm before (**A**–**C**) and after in vitro capacitation (**D**–**F**). Scale bar = 15 μm.

Western blot analysis of protein extracts prepared from human sperm before and after induction of in vitro capacitation showed that both Gα subunits are expressed in freshly prepared uncapacitated as well as in in vitro capacitated sperm (Figure 6). However, for α-gustducin, immunoreactivity was not only restricted to the predicted theoretical band of about −36 kDa, but was also visible for two additional immunopositive bands at about −50 kDa and −65 kDa, respectively, both in uncapacitated and capacitated sperm. Regarding α-transducin in noncapacitated sperm, an additional band of −40 kDa was observable beside the predicted band with a molecular mass of about −50 kDa. However, in capacitated sperm, an extra α-transducin-reactive band at a higher molecular mass (−75 kDa) was detectable. The quantitative analysis of main spots enabled us to confirm that transducin is present at a higher level than gustducin in both untreated and capacitated sperm (Supplementary Figure S2). According to these results, the cellular localization of α-gustducin and α-transducin has been evaluated in human sperm before and after in vitro capacitation. Figure 6 (A–C, left panel) shows a representative image of α-gustducin staining in freshly prepared uncapacitated sperm, characterized by an acrosomal signal, associated with a positive labelling of the sperm’s pericentriolar region and the tail. After the capacitation process, the Gα protein disappears from the acrosomal region, whereas the staining in the region of the postnuclear cup and along the entire tail was found to increase. This observation led to the suggestion that the process of secondary maturation of sperm seems to induce a subcellular translocation of α-gustducin to another sub-compartment of the cell Figure 6 (D–F, left panel), a reorganization also noticed for individual TAS2Rs (see above). Anyway, we cannot exclude that this specific pattern may be due to the occurrence of the acrosome reaction (as highlighted by PSA staining).

Comparing next the cellular distribution of α-transducin in uncapacitated sperm, one gets the impression that α-transducin was much stronger expressed than α-gustducin, thus confirming the results obtained by ddPCR (Figure 5). The bride fluorescence signals were visible in the equatorial region of the head, in the postnuclear region, and in the whole tail. In addition, it was also observable that capacitation induces a reorganization of α-transducin: whereas uncapacitated sperm showed a pronounced staining of the equatorial segment and a labeling along the entire tail (Figure 6A,C), fluorescence signals in capacitated sperm were much more concentrated in the region of the postnuclear region (Figure 6D,F).

Although α-gustducin was the first protein to be identified in taste cells [21], its role in taste signal transduction is still not fully understood; and this unclear function of α-gustducin is even more true for male germ cells. Both α-gustducin and α-transducin are expected to activate a phosphodiesterase (PDE) [37], thereby decreasing the intracellular level of the second messenger cAMP. By contrast, the β/γ complex of the activated G-protein complex was shown to stimulate phospholipase C-β2 (PLCβ2), leading to the breakdown of phosphatidylinositol bisphosphate (PIP_2_) into diacylglycerol (DAG) and inositol trisphosphate (IP_3_) [38,39]. IP_3_ subsequently triggers the release of calcium from the endoplasmic reticulum, thereby leading to the activation of the transient receptor potential protein melastatin-5 (TRPM5) [40,41,42].

In light of this established dual bitter taste transduction process in taste cells, and in order to assess whether this signaling pathway may also work in human sperm, we also evaluated the expression of *PDE4A, PLCβ2,* and *TRPM5* in ejaculated sperm using ddPCR.

**Figure 7:**
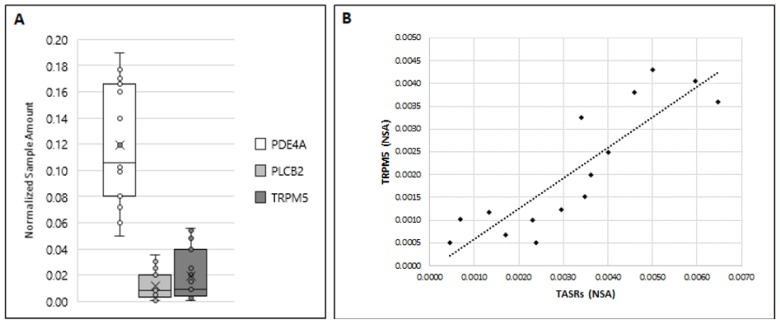
(**A**) Expression of *PDE4A*, *PLCB2*, and *TRPM5* in human ejaculated sperm. Graphical diagram is plotted as box–whisker plots, where boxes show the interquartile range with median and mean values, and whiskers represent min and max confidence intervals. (**B**) Correlation between *TASRs* and *TRPM5* (*r* = 0.87; *p* < 0.001) was determined by using Spearman’s correlation analysis.

The presented data in Figure 7 indicate an expression variability of the three analyzed genes of downstream components of signaling pathways in mature sperm. Although all tested sperm samples were found to express the three genes, their relative amount was significantly different, with mRNA for *PDE4A* being present at the highest level, whereas number of mRNA copies for *PLCβ2* and *TRPM5* were lower and almost equal (Figure 7A). Interestingly, global expression of members of the *TAS2Rs* family and the calcium-gated channel *TRPM5* showed a significant correlation in the number of amplified copies (*r* = 0.87) (Figure 7B).

## 4. Discussion

This study, for the first time, provides evidence for the expression of members of the bitter TAS2Rs family in ejaculated human sperm cells as well as in testicular tissue. In addition, expression of single components of the taste signal transduction cascade, such as the α-subtypes gustducin and transducin and the effector enzymes PDE4A, PLCβ2, and TRPM5, was observed in testis tissue and ejaculated spermatozoa.

Although in the first instance TAS2Rs were considered to exclusively have gustatory function, numerous studies addressed nongustatory activities for these sensory receptor proteins [43,44,45,46,47]. Indeed, TAS2Rs have been reported to be expressed in the respiratory system, where they affect respiratory functions in response to noxious stimuli, as well as in the gastrointestinal tract, where TAS2Rs activate metabolic and digestive signaling pathways upon binding of distinct bitter compounds [46]. In light of these pleiotropic activities, it has been supposed that taste receptors in the male reproductive system may be involved in controlling male fertility, probably during the processes of spermatogenesis, epididymal sperm maturation, sperm’s migration through the female genital tract, and/or upon final sperm egg fusion [48]. This is quite conceivable because during their transit through the epididymis, but also during the sperm journey through the vagina, uterus and the fallopian tubes, sperm cells are exposed to different concentrations of possible external ligands for taste receptors. This comprises different structurally unrelated hormones (e.g., proteins, peptides, or steroids), single amino acids, carbohydrates, changes in pH and bicarbonate, but also encountered toxins. Therefore, it has been suggested that taste receptor proteins and the coupled signal transduction cascade might be functionally operative in the perception of different biochemical stimuli, thereby driving sperm maturation, motility, chemotaxis, and probably also final fertilization [6,8,26,48,49].

One observation confirming this hypothesis comes from genetic studies; in this regard, polymorphic variants in taste receptors are often functional, and several gene polymorphisms have been proposed as modulators of spermatogenetic process efficiency, leading in some cases to impaired sperm production [50,51,52]. Recently, our research group definitively demonstrated the role of the genetic variability of taste receptors in human male infertility [53]. In light of this genetic correlation, we selected several TAS2Rs, whose expression has been tested in the present study, both in human sperm and in testis tissue.

Our results demonstrate that mRNA copies from different bitter taste receptor subtypes are detectable in a variable percentage of analyzed samples, ranging from 73% to 80%. This observation is in agreement with studies in taste buds [30] and shows that each sperm cell expresses, at a different level, a distinct subset of taste receptor subtypes, thus indicating a high heterogeneity of TAS2Rs expression at the cellular level of germ cells. Remarkably, for gustatory cells, it has been demonstrated that not all cells express all members of the bitter receptor family 2 in order to discriminate different bitter compounds [20,30]. Although we currently do not know which TAS2 receptor subtypes are not expressed in individual sperm cells, the important question arises of how the expression of particular human bitter taste receptors in sperm may be finely tuned to possibly detect a limited subset of bitter stimuli, a question to also ask for taste cells on the tongue [30].

Moreover, the low expression level we highlighted in human testis with the ultrasensitive approach of ddPCR match data in the mouse model system, which shows that the mouse genes *Tas2r137*, *Tas2r108,* and *Tas2r140* (orthologous genes of *TAS2R3, TAS2R4,* and *TAS2R14,* respectively) are only rarely expressed in mouse testis, with a transcript copy number below 100 [26]. Although TAS2R19 and TAS2R43 do not have mouse orthologues [54], our observation led to the suggestion of an identical and thus species-spanning expression level of individual bitter taste receptor subtypes in sperm.

Interestingly, we also provide evidence for a redistribution of TAS2Rs within the sperm after in vitro capacitation. For example, capacitated sperm was found to be concentrated in the midpiece of the flagellum, whereas uncapacitated sperm TAS2R43, in addition to its expression in the entire flagellum, was also localized to the pericentriolar region. The sequestering of distinct proteins and lipids to a specific subcellular region within sperm is a strategy often adopted during secondary sperm maturation within the female genital tract. For example, it is known that a lateral redistribution of seminolipids [55] as well as a subcellular reorganization of proteins involved in acrosome reaction and in oocyte’s binding occur during sperm capacitation [56,57]. Although the functional role of bitter taste receptors in sperm is currently not clear, the detected different glycosylation level of individual TAS2Rs may indicate that this covalent modification could probably be operative in the observed capacitation-dependent redistribution and subsequent targeting of receptors to a distinct site of the cell [58,59].

The present study also demonstrates the expression of α-gustducin and α-transducin in ejaculated human sperm cells. α-gustducin has already been observed to be expressed during mouse spermatogenesis [22], together with the entire bitter taste transduction cascade, thus suggesting a detection of chemical substances solved in the lumen of the seminiferous tubules [7]. α-gustducin, together with α-transducin, has also been detected in boar testicular tissue and ejaculated spermatozoa [49]. Since capacitation actually renders sperm competent to fertilize an egg, we herein also investigated the expression of both G proteins α-subtypes in uncapacitated and capacitated human sperm. Interestingly, performing indirect immunofluorescence analysis, a maturation-dependent subcellular redistribution of the two G proteins was uncovered: upon capacitation, α-gustducin and α-transducin both disappear from the anterior part of the head, and emerge at a higher concentration in the postnuclear region and in the tail. This phenomenon of translocation may be relevant for several physiologically significant processes, such as acrosomal reaction, *zona pellucida* penetration, as well as hyperactivated sperm motility [60,61]. In addition, Western blot analysis showed different G protein isoforms in capacitated spermatozoa compared with freshly isolated sperm, which probably reflects post-translational modifications, such as myristoylation and farnesylation [62], proteolytic cleavage, phosphorylation, or nucleotide exchange (GDP to GTP), as has been described for transducin in the retina [63]. The appearance of the larger immunoreactive band for α-transducin in capacitated sperm might be due to the formation of insoluble complexes [64] or the result of an association with G protein regulating proteins, such as GAPs, GEFs, or GDIs [65]. Although the exact functional relevance of a possible post-translational modification of gustducin and transducin or an interaction with regulatory proteins is currently not known, one may suggest that this alteration could reflect a different activation stage of the two G protein subtypes in freshly prepared sperm versus fully capacitated spermatozoa or might be important for the detected capacitation-dependent redistribution.

In the presented manuscript, we also demonstrated for the first time the expression of *TRPM5* in human testis tissue as well as in sperm and additionally provide evidence for a significant correlation between *TRPM5* and *TAS2Rs* mRNA levels. TRPM5, the common downstream element for sweet, umami, as well as in bitter taste transduction [66], seems to have a more restricted expression profile, if compared with other members of the huge family of TRP channel proteins [67]. Another unique feature of TRPM5 and its taste counterpart TRPM4 [68] is that, unlike most other members of the TRP family, these channels are not permeable for calcium but instead allow sodium entry into the cell. However, calcium represents the key regulator of their activity since both channel proteins are activated by a rise in intracellular calcium [69]. Many mechanisms involved in key sperm physiological responses, essential for successful fertilization, such as chemotaxis, hyperactivated motility, and the acrosome reaction are accomplished by calcium and coupled signaling pathways [70,71]. Therefore, calcium homeostasis is of primary importance for proper sperm physiology, as also demonstrated for the essential presence of the sperm-specific calcium channel CatSper [72]. Remarkably, species not expressing CatSper also exhibit calcium influx, thus suggesting the existence of other mechanisms triggering increase in calcium in sperm cells [73]. In this context, it is interesting to mention that TAS2R4, detected to exhibit the highest expression level of examined bitter taste receptors in human sperm, plays an important role in the activation of a signaling pathway that results in elevation of cytosolic calcium. Indeed, its agonist quinine, through the activation of a chimeric Gα16 protein fused to 44 amino acids of gustducin (G16/gust44), inactivates Rac1, a member of the Rho family of small GTPases that regulate actin cytoskeleton reorganization [74]. Therefore, considering the main role of the actin cytoskeleton network on acrosome molding and development, and its persistence in the subacrosomal space of mature mammalian sperm, including humans [75], an important role of TAS2R4 in male acrosome ontogeny and physiology may be hypothesized.

Moreover, the herein observed significant correlation between TAS2Rs and TRPM5 expression in human sperm described for the first time may provide evidence for a functional link between the two signaling molecules in controlling male gamete physiology. Since it is known that bitter compounds, such as caffeine, are able to induce sperm hyperactivation [76], one might speculate that bitter compounds can control sperm motility by a TAS2R-induced TRPM5 stimulation, which, due to its sodium selectivity, can then induce depolarization of sperm. The subsequent change in membrane potential can then directly affect the driving force for cations, such as calcium, but may also trigger the activity of voltage-gated calcium channels [66].

However, it also cannot be excluded that TAS2Rs in sperm may have a more defensive and protective function, as clearly demonstrated for gustatory cells, where they serve as warning sensors against the ingestion of toxic food compounds [77]. In this context, it is essential to mention recent observation showing that animals able to avoid noxious foods by using bitter taste receptors have a highly efficient spermatogenesis and, as a consequence, may produce more offspring [78].

Even if future research is necessary to determine the actual contribution of TAS2Rs for proper sperm physiology, our discovery of TAS2Rs expression in human testis and sperm may pave the way to better understand the molecular mechanisms underlying the communication between environmental activators or toxicants and male germ cells, as well as male gametes.

## Figures and Tables

**Table 1:** PrimePCR EvaGreen Assay, specific for Droplets Digital Polymerase Chain Reaction. cAMP, Cyclic adenosine monophosphate.

Target Genes	Acronym	ID Assay
Taste receptor, type 2, member 3	*TAS2R3*	dHsaEG5003946
Taste receptor, type 2, member 4	*TAS2R4*	dHsaEG5003947
Taste receptor, type 2, member 14	*TAS2R14*	dHsaEG5004113
Taste receptor, type 2, member 19	*TAS2R19*	dHsaEG5003736
Taste receptor, type 2, member 43	*TAS2R43*	dHsaEG5004567
Transducin Alpha-1 Chain	*GNAT1*	dHsaEG5000400
Gustducin Alpha-3 Chain	*GNAT3*	dHsaEG5024588
Phosphodiesterase 4A, cAMP-specific	*PDE4A*	dHsaEG5014540
Phospholipase C, beta 2	*PLCB2*	dHsaEG5015173
Transient receptor potential channel, subfamily M, member 5	*TRPM5*	dHsaEG5001934
**Reference Genes**	**Acronym**	**ID Assay**
Peptidylprolyl isomerase B (cyclophilin B)	*PPIB*	dHsaEG5022088
Glyceraldehyde-3-phosphate dehydrogenase	*GAPDH*	dHsaEG5006642
Actin, beta	*ACT-B*	dHsaEG5188254
Beta-2-microglobulin	*B2M*	dHsaEG5020739

## Supplementary Materials

The following are available online at https://www.mdpi.com/2077-0383/9/1/264/s1, Supplementary Table S1: List of antibodies used in this study; Supplementary Figure S1: Computer-assisted semi-quantitative analysis of the overall relative intensity of the bands from western blot of TAS2R3, TAS2R4, TAS2R14, TAS2R19 and TAS2R43 in extracts of human sperm before (BS) and after in vitro capacitation (CS); Supplementary Figure S2: Computer-assisted semi-quantitative analysis of the overall relative intensity of the bands from western blot of α-Gustducin and α-Transducin in extracts of human sperm before (BS) and after in vitro capacitation (CS).

## References

[1] Hoon M.A., Adler E., Lindemeier J., Battey J.F., Ryba N.J., Zuker C.S. (1999). Putative mammalian taste receptors: A class of taste-specific GPCRs with distinct topographic selectivity. Cell.

[2] Lee S.-J., Depoortere I., Hatt H. (2019). Therapeutic potential of ectopic olfactory and taste receptors. Nat. Rev. Drug Discov..

[3] Shah A.S., Ben-Shahar Y., Moninger T.O., Kline J.N., Welsh M.J. (2009). Motile cilia of human airway epithelia are chemosensory. Science.

[4] Fujita T. (1991). Taste cells in the gut and on the tongue. Their common, paraneuronal features. Physiol. Behav..

[5] Ren X., Zhou L., Terwilliger R., Newton S.S., de Araujo I.E. (2009). Sweet taste signaling functions as a hypothalamic glucose sensor. Front. Integr. Neurosci..

[6] Li F. (2013). Taste perception: From the tongue to the testis. Mol. Hum. Reprod..

[7] Li F., Zhou M. (2012). Depletion of bitter taste transduction leads to massive spermatid loss in transgenic mice. Mol. Hum. Reprod. Med..

[8] Meyer D., Voigt A., Widmayer P., Borth H., Huebner S., Breit A., Marschall S., de Angelis M.H., Boehm U., Meyerhof W. (2012). Expression of Tas1 taste receptors in mammalian spermatozoa: Functional role of Tas1r1 in regulating basal Ca^2+^ and cAMP concentrations in spermatozoa. PLoS ONE.

[9] Mosinger B., Redding K.M., Parker M.R., Yevshayeva V., Yee K.K., Dyomina K., Li Y., Margolskee R.F. (2013). Genetic loss or pharmacological blockade of testes-expressed taste genes causes male sterility. Proc. Natl. Acad. Sci. USA.

[10] Ishimaru Y., Matsunami H. (2009). Transient receptor potential (TRP) channels and taste sensation. J. Dent. Res..

[11] Liman E.R., Zhang Y.V., Montell C. (2014). Peripheral coding of taste. Neuron.

[12] Max M., Shanker Y.G., Huang L., Rong M., Liu Z., Campagne F., Weinstein H., Damak S., Margolskee R.F. (2001). Tas1r3, encoding a new candidate taste receptor, is allelic to the sweet responsiveness locus Sac. Nat. Genet..

[13] Montmayeur J.P., Matsunami H. (2002). Receptors for bitter and sweet taste. Curr. Opin. Neurobiol..

[14] Bachmanov A.A., Beauchamp G.K. (2007). Taste receptor genes. Annu. Rev. Nutr..

[15] Chandrashekar J., Hoon M.A., Ryba N.J.P., Zuker C.S. (2006). The receptors and cells for mammalian taste. Nature.

[16] Behrens M., Briand L., de March C.A., Matsunami H., Yamashita A., Meyerhof W., Weyand S. (2018). Structure–Function Relationships of Olfactory and Taste Receptors. Chem. Senses.

[17] Roper S.D., Chaudhari N. (2017). Taste buds: Cells, signals and synapses. Nat. Rev. Neurosci..

[18] Roper S.D., Chaudhari N. (2009). Processing umami and other tastes in mammalian taste buds. Ann. N. Y. Acad. Sci..

[19] Adler E., Hoon M.A., Mueller K.L., Chandrashekar J., Ryba N.J., Zuker C.S. (2000). A novel family of mammalian taste receptors. Cell.

[20] Matsunami H., Montmayeur J.P., Buck L.B. (2000). A family of candidate taste receptors in human and mouse. Nature.

[21] McLaughlin S.K., McKinnon P.J., Margolskee R.F. (1992). Gustducin is a taste-cell-specific G protein closely related to the transducins. Nature.

[22] Fehr J., Meyer D., Widmayer P., Borth H.C., Ackermann F., Wilhelm B., Gudermann T., Boekhoff I. (2007). Expression of the G-protein alpha-subunit gustducin in mammalian spermatozoa. J. Comp. Physiol. A Neuroethol. Sens. Neural. Behav. Physiol..

[23] McLaughlin S.K., McKinnon P.J., Spickofsky N., Danho W., Margolskee R.F. (1994). Molecular cloning of G proteins and phosphodiesterases from rat taste cells. Physiol. Behav..

[24] Stone L.M., Barrows J., Finger T.E., Kinnamon S.C. (2007). Expression of T1Rs and gustducin in palatal taste buds of mice. Chem. Senses.

[25] Voigt A., Hübner S., Lossow K., Hermans-Borgmeyer I., Boehm U., Meyerhof W. (2012). Genetic labeling of Tas1r1 and Tas2r131 taste receptor cells in mice. Chem. Senses.

[26] Xu J., Cao J., Iguchi N., Riethmacher D., Huang L. (2013). Functional characterization of bitter-taste receptors expressed in mammalian testis. Mol. Hum. Reprod..

[27] Glendinning J.I. (1994). Is the bitter rejection response always adaptive?. Physiol. Behav..

[28] Hofmann T. (2009). Identification of the key bitter compounds in our daily diet is a prerequisite for the understanding of the hTAS2R gene polymorphisms affecting food choice. Ann. N. Y. Acad. Sci..

[29] Maehashi K., Huang L. (2009). Bitter peptides and bitter taste receptors. Cell. Mol. Life Sci. CMLS.

[30] Behrens M., Foerster S., Staehler F., Raguse J.-D., Meyerhof W. (2007). Gustatory expression pattern of the human TAS2R bitter receptor gene family reveals a heterogenous population of bitter responsive taste receptor cells. J. Neurosci. Off. J. Soc. Neurosci..

[31] World Health Organization (2010). WHO Laboratory Manual for the Examination and Processing of Human Semen.

[32] Evaluation of Mitochondrial Respiratory Efficiency during in Vitro Capacitation of Human Spermatozoa-Stendardi-2011-International Journal of Andrology-Wiley Online Library. https://onlinelibrary.wiley.com/doi/full/10.1111/j.1365-2605.2010.01078.x.

[33] Cleavage of Structural Proteins during the Assembly of the Head of Bacteriophage T4|Nature. https://www.nature.com/articles/227680a0.

[34] Reichling C., Meyerhof W., Behrens M. (2008). Functions of human bitter taste receptors depend on N-glycosylation. J. Neurochem..

[35] Huang L., Shanker Y.G., Dubauskaite J., Zheng J.Z., Yan W., Rosenzweig S., Spielman A.I., Max M., Margolskee R.F. (1999). Ggamma13 colocalizes with gustducin in taste receptor cells and mediates IP3 responses to bitter denatonium. Nat. Neurosci..

[36] Kim M.-R., Kusakabe Y., Miura H., Shindo Y., Ninomiya Y., Hino A. (2003). Regional expression patterns of taste receptors and gustducin in the mouse tongue. Biochem. Biophys. Res. Commun..

[37] Hoon M.A., Northup J.K., Margolskee R.F., Ryba N.J. (1995). Functional expression of the taste specific G-protein, alpha-gustducin. Biochem. J..

[38] Rössler P., Kroner C., Freitag J., Noè J., Breer H. (1998). Identification of a phospholipase C beta subtype in rat taste cells. Eur. J. Cell Biol..

[39] Rössler P., Boekhoff I., Tareilus E., Beck S., Breer H., Freitag J. (2000). G protein betagamma complexes in circumvallate taste cells involved in bitter transduction. Chem. Senses.

[40] Pérez C.A., Huang L., Rong M., Kozak J.A., Preuss A.K., Zhang H., Max M., Margolskee R.F. (2002). A transient receptor potential channel expressed in taste receptor cells. Nat. Neurosci..

[41] Ruiz-Avila L., McLaughlin S.K., Wildman D., McKinnon P.J., Robichon A., Spickofsky N., Margolskee R.F. (1995). Coupling of bitter receptor to phosphodiesterase through transducin in taste receptor cells. Nature.

[42] Zhang Z., Zhao Z., Margolskee R., Liman E. (2007). The transduction channel TRPM5 is gated by intracellular calcium in taste cells. J. Neurosci. Off. J. Soc. Neurosci..

[43] Kinnamon S.C. (2012). Taste receptor signaling-from tongues to lungs. Acta Physiol. Oxf. Engl..

[44] Rozengurt E. (2006). Taste receptors in the gastrointestinal tract. I. Bitter taste receptors and alpha-gustducin in the mammalian gut. Am. J. Physiol. Gastrointest. Liver Physiol..

[45] Elliott R.A., Kapoor S., Tincello D.G. (2011). Expression and distribution of the sweet taste receptor isoforms T1R2 and T1R3 in human and rat bladders. J. Urol..

[46] Behrens M., Meyerhof W. (2010). Oral and extraoral bitter taste receptors. Results Probl. Cell Differ..

[47] Shaik F.A., Singh N., Arakawa M., Duan K., Bhullar R.P., Chelikani P. (2016). Bitter taste receptors: Extraoral roles in pathophysiology. Int. J. Biochem. Cell Biol..

[48] Luddi A., Governini L., Wilmskötter D., Gudermann T., Boekhoff I., Piomboni P. (2019). Taste Receptors: New Players in Sperm Biology. Int. J. Mol. Sci..

[49] Spinaci M., Bucci D., Mazzoni M., Giaretta E., Bernardini C., Vallorani C., Tamanini C., Clavenzani P., Galeati G. (2014). Expression of α-gustducin and α-transducin, G proteins coupled with taste receptors, in boar sperm. Theriogenology.

[50] Aston K.I., Krausz C., Laface I., Ruiz-Castané E., Carrell D.T. (2010). Evaluation of 172 candidate polymorphisms for association with oligozoospermia or azoospermia in a large cohort of men of European descent. Hum. Reprod. Oxf. Engl..

[51] Plaseski T., Noveski P., Popeska Z., Efremov G.D., Plaseska-Karanfilska D. (2012). Association study of single-nucleotide polymorphisms in FASLG, JMJDIA, LOC203413, TEX15, BRDT, OR2W3, INSR, and TAS2R38 genes with male infertility. J. Androl..

[52] Siasi E., Aleyasin A. (2016). Four Single Nucleotide Polymorphisms in INSR, SLC6A14, TAS2R38, and OR2W3 Genes in Association with Idiopathic Infertility in Persian Men. J. Reprod. Med..

[53] Gentiluomo M., Crifasi L., Luddi A., Locci D., Barale R., Piomboni P., Campa D. (2017). Taste receptor polymorphisms and male infertility. Hum. Reprod. Oxf. Engl..

[54] Luo M., Ni K., Jin Y., Yu Z., Deng L. (2019). Toward the Identification of Extra-Oral TAS2R Agonists as Drug Agents for Muscle Relaxation Therapies via Bioinformatics-Aided Screening of Bitter Compounds in Traditional Chinese Medicine. Front. Physiol..

[55] Goto-Inoue N., Hayasaka T., Zaima N., Setou M. (2009). The specific localization of seminolipid molecular species on mouse testis during testicular maturation revealed by imaging mass spectrometry. Glycobiology.

[56] Boerke A., Tsai P.S., Garcia-Gil N., Brewis I.A., Gadella B.M. (2008). Capacitation-dependent reorganization of microdomains in the apical sperm head plasma membrane: Functional relationship with zona binding and the zona-induced acrosome reaction. Theriogenology.

[57] Ackermann F., Zitranski N., Heydecke D., Wilhelm B., Gudermann T., Boekhoff I. (2008). The Multi-PDZ domain protein MUPP1 as a lipid raft-associated scaffolding protein controlling the acrosome reaction in mammalian spermatozoa. J. Cell. Physiol..

[58] Lanctôt P.M., Leclerc P.C., Escher E., Guillemette G., Leduc R. (2006). Role of N-glycan-dependent quality control in the cell-surface expression of the AT1 receptor. Biochem. Biophys. Res. Commun..

[59] Michineau S., Muller L., Pizard A., Alhenc-Gélas F., Rajerison R.M. (2004). N-linked glycosylation of the human bradykinin B2 receptor is required for optimal cell-surface expression and coupling. Biol. Chem..

[60] Zitranski N., Borth H., Ackermann F., Meyer D., Vieweg L., Breit A., Gudermann T., Boekhoff I. (2010). The “acrosomal synapse”: Subcellular organization by lipid rafts and scaffolding proteins exhibits high similarities in neurons and mammalian spermatozoa. Commun. Integr. Biol..

[61] Gadella B.M. (2008). Sperm membrane physiology and relevance for fertilization. Anim. Reprod. Sci..

[62] Gao Y., Erickson J.W., Cerione R.A., Ramachandran S. (2019). Purification of the Rhodopsin-Transducin Complex for Structural Studies. Methods Mol. Biol. Clifton NJ.

[63] Calvert P.D., Krasnoperova N.V., Lyubarsky A.L., Isayama T., Nicoló M., Kosaras B., Wong G., Gannon K.S., Margolskee R.F., Sidman R.L. (2000). Phototransduction in transgenic mice after targeted deletion of the rod transducin alpha -subunit. Proc. Natl. Acad. Sci. USA.

[64] Von Buchholtz L., Elischer A., Tareilus E., Gouka R., Kaiser C., Breer H., Conzelmann S. (2004). RGS21 is a novel regulator of G protein signalling selectively expressed in subpopulations of taste bud cells. Eur. J. Neurosci..

[65] Siderovski D.P., Willard F.S. (2005). The GAPs, GEFs, and GDIs of heterotrimeric G-protein alpha subunits. Int. J. Biol. Sci..

[66] Guinamard R., Sallé L., Simard C., Islam M.d.S. (2011). The Non-selective Monovalent Cationic Channels TRPM4 and TRPMIn. Transient Receptor Potential Channels.

[67] Kusumakshi S., Voigt A., Hübner S., Hermans-Borgmeyer I., Ortalli A., Pyrski M., Dörr J., Zufall F., Flockerzi V., Meyerhof W. (2015). A Binary Genetic Approach to Characterize TRPM5 Cells in Mice. Chem. Senses.

[68] Dutta Banik D., Martin L.E., Freichel M., Torregrossa A.-M., Medler K.F. (2018). TRPM4 and TRPM5 are both required for normal signaling in taste receptor cells. Proc. Natl. Acad. Sci. USA.

[69] Prawitt D., Monteilh-Zoller M.K., Brixel L., Spangenberg C., Zabel B., Fleig A., Penner R. (2003). TRPM5 is a transient Ca2+-activated cation channel responding to rapid changes in [Ca2+]i. Proc. Natl. Acad. Sci. USA.

[70] Weissgerber P., Kriebs U., Tsvilovskyy V., Olausson J., Kretz O., Stoerger C., Vennekens R., Wissenbach U., Middendorff R., Flockerzi V. (2011). Male fertility depends on Ca^2^+ absorption by TRPV6 in epididymal epithelia. Sci. Signal..

[71] Brown S.G., Publicover S.J., Barratt C.L.R., Martins da Silva S.J. (2019). Human sperm ion channel (dys) function: Implications for fertilization. Hum. Reprod. Update.

[72] Lishko P.V., Mannowetz N. (2018). CatSper: A Unique Calcium Channel of the Sperm Flagellum. Curr. Opin. Physiol..

[73] Lishko P.V., Kirichok Y., Ren D., Navarro B., Chung J.-J., Clapham D.E. (2012). The Control of Male Fertility by Spermatozoan Ion Channels. Annu. Rev. Physiol..

[74] Sidhu C., Jaggupilli A., Chelikani P., Bhullar R.P. (2017). Regulation of Rac1 GTPase activity by quinine through G-protein and bitter taste receptor T_2_R_4_. Mol. Cell. Biochem..

[75] Breitbart H., Cohen G., Rubinstein S. (2005). Role of actin cytoskeleton in mammalian sperm capacitation and the acrosome reaction. Reprod. Camb. Engl..

[76] Colás C., Cebrián-Pérez J.A., Muiño-Blanco T. (2010). Caffeine induces ram sperm hyperactivation independent of cAMP-dependent protein kinase. Int. J. Androl..

[77] Antinucci M., Risso D. (2017). A Matter of Taste: Lineage-Specific Loss of Function of Taste Receptor Genes in Vertebrates. Front. Mol. Biosci..

[78] Leonard W.R. (2002). Food for thought. Dietary change was a driving force in human evolution. Sci. Am..

