# Supplementary material for: Expression of Taste Receptor 2 Subtypes in Human Testis and Sperm
Source: J Clin Med. 2020 Jan 18;9(1):264. doi: 10.3390/jcm9010264 (PMC7019805; doi:10.3390/jcm9010264)
Supplement: Supplementary file 1 [file jcm-09-00264-s001.pdf]

Supplementary Table S1. List of antibodies used in this study.

Antigen	*RRID*	*Donor species*	*Dilution*	
			IF	*WB*
Primary antibodies				
**GAPDH**	AB_10611262	Goat	/	1:500
α-gustducin	AB_673678	Rabbit	1:100	1:500
α-transducin	AB_2279097	Rabbit	1:100	1:500
TAS2R3	AB_2556263	Rabbit	1:100	1:500
TAS2R4	AB_1049824	Goat	1:100	1:500
TAS2R14	AB_11009211	Rabbit	1:100	1:500
TAS2R19	AB_962285	Rabbit	1:300	1:1000
TAS2R43	AB_2201092	Rabbit	1:300	1:1000
Secondary antibodies				
Anti-Rabbit-IgG FITC	AB_259430	Rabbit	1:500	1:100
Anti-Goat-IgG HRP	AB_92411	Rabbit	/	1:7500
Anti-Rabbit-IgG HRP	AB_11212848	Goat	/	1:2000

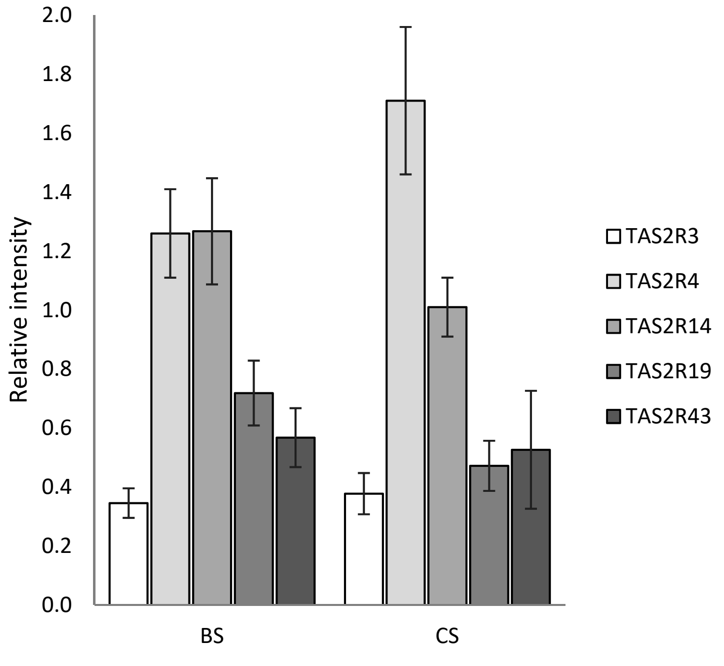

Supplementary Figure S1. Computer-assisted semi-quantitative analysis of the overall relative intensity of the bands from western blot of TAS2R3, TAS2R4, TAS2R14, TAS2R19 and TAS2R43 in extracts of human sperm before (BS) and after *in vitro* capacitation (CS). The intensity was measured (pixel/mm^2) and then normalized relative to GAPDH. Values are expressed as mean ± SD.

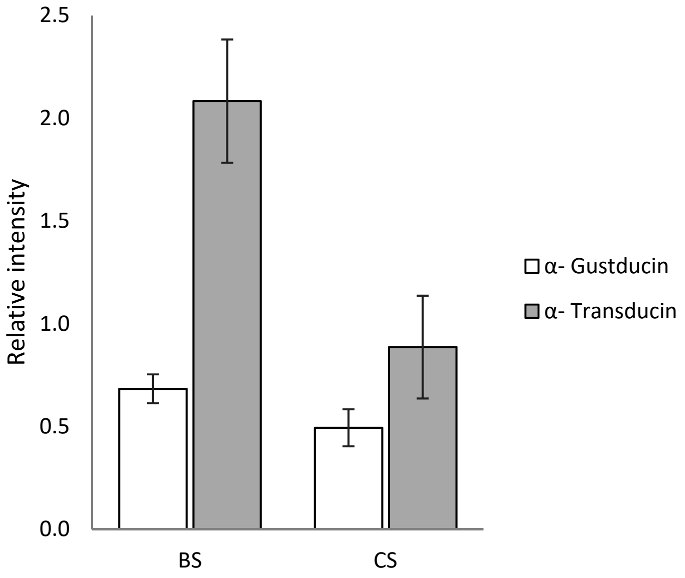

Supplementary Figure S2. Computer-assisted semi-quantitative analysis of the overall relative intensity of the bands from western blot of α-Gustducin and α-Transducin in extracts of human sperm before (BS) and after *in vitro* capacitation (CS). The intensity was measured (pixel/mm^2) and then normalized relative to GAPDH. Values are expressed as mean ± SD.
